# Performance of cryptogenic new onset refractory status epilepticus score in a Brazilian cohort after testing for antineuronal antibodies with tissue‐based and cell‐based assays

**DOI:** 10.1111/epi.18374

**Published:** 2025-05-02

**Authors:** João Henrique Fregadolli Ferreira, Anna Maria Gomes, Ana Carolina Zetehaku, Taissa Ferrari Marinho, Fabio Fieni Toso, Caio César Diniz Disserol, Mariana Braatz Krueger, Mara Lúcia Schmitz Ferreira Santos, Aline Freire Borges Juliano, Tércio Luz Barbosa, Letícia Januzi de Almeida Rocha, Fernando Tenório Gameleira, Renata Barbosa Paolilo, Vanessa Daccach, João Pedro Izidoro Gomes, Katia Lin, Adaucto Wanderley da Nóbrega, Emily Lima Marmentini, Eduardo Ferracioli Fusão, Anderson de Cuffa, Georgia Lelis Aranha Tavares, Ronaldo Maciel Dias, Sabrina Stephanie Lana Diniz, Fábio Siquineli, Pedro Braga‐Neto, Paulo Ribeiro Nobrega, Lorena Pitombeira Sanders, Fernanda Martins Maia Carvalho, Renata Brasileiro Reis Pereira, Eduardo Sousa de Melo, Adélia Maria de Miranda Henriques‐Souza, Clecio de Oliveira Godeiro, Omar Gurrola Arambula, Filipe Nolasco de Souza e Silva, Karla Oliveira Couto, Dayane Danieli, Katia Werneck Seitz, Alessandra Dellavance, Verena Endmayr, Romana Höftberger, Luis Eduardo Coelho Andrade, Luis Otavio Caboclo, Livia Almeida Dutra

**Affiliations:** ^1^ Instituto do Cérebro, Hospital Israelita Albert Einstein São Paulo Brazil; ^2^ Hospital de Clínicas da Universidade Federal do Paraná Curitiba Brazil; ^3^ Instituto de Neurologia de Curitiba Curitiba Brazil; ^4^ Hospital Infantil Albert Sabin Fortaleza Brazil; ^5^ Hospital Pequeno Príncipe Curitiba Brazil; ^6^ Hospital Universitário da Universidade Federal do Maranhão São Luíz Brazil; ^7^ Universidade Federal do Piauí—Campus Senador Helvídio Nunes de Barros Picos Brazil; ^8^ Hospital Universitário Professor Alberto Antunes da Faculdade de Medicina da Universidade Federal do Alagoas, EBSERH Maceió Brazil; ^9^ Instituto da Criança Hospital das Clínicas da Faculdade de Medicina da Universidade de São Paulo São Paulo Brazil; ^10^ Faculdade de Medicina de Ribeirão Preto Universidade de São Paulo Ribeirão Preto Brazil; ^11^ Universidade Federal de Santa Catarina Florianópolis Brazil; ^12^ Hospital Infantil Joana de Gusmão Florianópolis Brazil; ^13^ Hospital da UNIMED Grande Florianópolis Florianópolis Brazil; ^14^ Grupo Hospitalar Conceição Porto Alegre Brazil; ^15^ Hospital de Base de Brasília (IGESDF) Brasília Brazil; ^16^ Universidade Federal de Minas Gerais Belo Horizonte Brazil; ^17^ Hospital Santa Isabel Blumenau Brazil; ^18^ Departamento de Clínica Médica Universidade Federal do Ceará Fortaleza Brazil; ^19^ Hospital Universitário Walter Cantídio, Faculdade de Medicina Universidade Federal do Ceará Fortaleza Brazil; ^20^ Hospital Geral de Fortaleza Fortaleza Brazil; ^21^ Graduate Program in Medical Sciences, Universidade de Fortaleza Fortaleza Brazil; ^22^ Hospital da Criança de Brasília José Alencar Brasília Brazil; ^23^ Centro de Ciências Médicas, Universidade Federal de Pernambuco Recife Brazil; ^24^ Instituto de Medicina Integral Professor Fernando Figueira Recife Brazil; ^25^ Hospital da Restauração Recife Brazil; ^26^ Universidade Federal do Rio Grande do Norte Natal Brazil; ^27^ Universidade Estadual de Mato Grosso do Sul Dourados Brazil; ^28^ Hospital Santa Izabel Salvador Brazil; ^29^ Universidade Federal do Mato Grosso do Sul Campo Grande Brazil; ^30^ Hospital da Criança Augusta Muller Bohner (Materno–Infantil) Chapecó Brazil; ^31^ Grupo Fleury São Paulo Brazil; ^32^ Division of Neuropathology and Neurochemistry, Department of Neurology Medical University of Vienna Vienna Austria; ^33^ Comprehensive Center for Clinical Neurosciences and Mental Health Medical University of Vienna Vienna Austria; ^34^ Escola Paulista de Medicina Universidade Federal de São Paulo São Paulo Brazil

**Keywords:** autoimmune encephalitis, c‐NORSE score, epilepsy, febrile infection‐related epilepsy syndrome, new onset refractory status epilepticus, status epilepticus

## Abstract

**Objective:**

This study was undertaken to describe a case series of Brazilian new onset refractory status epilepticus (NORSE) patients and evaluate the sensitivity and specificity of a clinical score to predict cryptogenic etiology (c‐NORSE score) after autoimmune encephalitis (AE) testing.

**Methods:**

Thirty‐seven patients with NORSE from the Brazilian Autoimmune Encephalitis Network were investigated with brain magnetic resonance imaging (MRI), cerebrospinal fluid (CSF) analysis, and complementary testing with tissue‐based assays and cell‐based assays for antineuronal and antiglial antibodies (abs) in serum/CSF. Final diagnoses were compiled after chart review. Patients were allocated into two groups according to c‐NORSE score: (≥5) high score (HS) or (<5) low score (LS), and clinical variables were compared. c‐NORSE score sensitivity and specificity were calculated.

**Results:**

We found that 23 (62%) of the NORSE patients were children, 49% were female, 22% had AE, and 51% were classified as c‐NORSE. Eleven patients (30%) were allocated to the HS group and 26 (70%) to LS. Bilateral and symmetric MRI findings were more frequent in the HS group (HS 100% vs. LS 43%, *p* = .018), whereas memory or behavioral symptoms were less common (HS 27% vs. LS 89%, *p* = .001). All HS patients had c‐NORSE, whereas 69% of the LS patients had other diagnoses, such as AE (*n* = 8), herpes simplex virus encephalitis (*n* = 4), paraneoplastic encephalomyelitis (*n* = 1), acute disseminated encephalomyelitis (*n* = 1), and other causes (*n* = 4). The sensitivity of the c‐NORSE score for predicting cryptogenic cases was 57.9% (95% confidence interval [CI] = 36%–80%), and the specificity was 100% (95% CI = 84%–100%).

**Significance:**

In this cohort, AE was the most identified cause of NORSE, and 51% were cryptogenic. c‐NORSE score ≥ 5 had a specificity of 100% (95% CI = 84%–100%) for identifying cryptogenic cases, whereas a score < 5 indicates additional investigation should be ordered. Although the sensitivity of the c‐NORSE score was lower than previously reported, suggesting it may vary depending on complementary investigation, it is a useful tool for bedside NORSE evaluation in low‐income countries, where access to antineuronal abs is limited.

**Key points**

- Approximately 22% of NORSE patients have AE, when investigated with TBAs and CBAs for antineuronal antibodies and anti‐MOG.
- The sensitivity of the c‐NORSE score varies depending on the investigation performed and categorization of the final diagnosis.
- Anti‐MOG may be found in pediatric patients with NORSE.
- In patients with c‐NORSE score ≥ 5, the yield of antineuronal abs testing is low.
- A c‐NORSE score < 5 indicates that additional investigation is needed, including antineuronal abs.
- C‐NORSE score is a useful tool for bedside evaluation in low‐income countries, where access to antineuronal abs testing is limited.

## 1 INTRODUCTION

New onset refractory status epilepticus (NORSE) is a clinical presentation defined by the occurrence of refractory status epilepticus (RSE) in a patient without active epilepsy or other preexisting relevant neurological disorder, and without a clear acute or active structural, toxic, or metabolic cause.^1^ A subgroup of NORSE patients known as febrile infection‐related epilepsy syndrome (FIRES) present with fever starting between 2 weeks and 24 h prior to the onset of RSE.^1^ These rare syndromes belong to the same clinical entity; their incidence is currently unknown, and mortality rates may range from 10% to 30%.^2^, ^3^, ^4^

Most NORSE patients remain without an identifiable cause for the RSE, even after extensive investigation, and are termed cryptogenic NORSE (c‐NORSE).^5^ In pediatric cases, the rate of c‐NORSE may reach up to 87%.^6^ On the other hand, adult patients more frequently have an identifiable cause, the most common being autoimmune encephalitis (AE).^2^, ^7^ Accurate estimates on the frequency of AE among NORSE patients are lacking, as a systematic review of 1334 patients with NORSE and FIRES reported that antineuronal antibodies (abs) were performed in only 60.9%.^7^ Moreover, in previous adult NORSE series, techniques employed for antineuronal ab detection were scarcely reported,^2^, ^8^, ^9^, ^10^, ^11^, ^12^, ^13^, ^14^, ^15^ and those reported were rarely aligned with the current testing recommendations.^16^, ^17^, ^18^, ^19^

Recommended AE testing requires using two complementary techniques, tissue‐based assay (TBA) and cell‐based assay (CBA), in paired samples of serum and cerebrospinal fluid (CSF).^18^, ^19^, ^20^ TBA is a rat brain immunohistochemical technique that allows detection of a variety of antineuronal abs and presents high sensitivity and specificity for diagnosis of AE.^21^ The morphologic pattern observed in the TBA suggests but does not specify the abs present in the sample. CBA uses indirect immunofluorescence in transfected cells that express only one specific neuronal surface antigen. CBAs are performed with live cells (usually in research laboratories) or with fixed cells, the latter being used in commercial kits.^21^ Preliminary data showed that fixed CBAs may lead to 9%–12% false negative results^21^, ^22^ and even false positive results in CSF,^23^ highlighting the importance of confirmatory results with TBA to prevent misdiagnosis.^16^, ^17^

Early recognition of NORSE etiology is essential for adequate management. According to an international consensus recommendation, first‐line immunological treatments such as intravenous immunoglobulin (IVIG), plasmapheresis, and steroids should be started for all patients within 72 h.^24^ Second‐line immunological therapies, however, depends on the suspected etiology of NORSE. Most experts agree that rituximab should be started when AE is highly suspicious or confirmed. Conversely, in c‐NORSE/FIRES, drugs targeting the innate immune pathways, such as interleukin (IL)‐1R or IL‐6R antagonists, should be initiated.^24^, ^25^

A clinically based score was developed and validated to predict c‐NORSE at the early stages of RSE.^26^, ^27^ In the pivotal study, the c‐NORSE score achieved sensitivity of 93.7% and specificity of 100%, and is based on six clinical features: (1) NORSE highly resistant to conventional antiseizure medication (ASM) treatment, (2) previously healthy individual before the onset of status epilepticus (SE), (3) presence of prodromal high fever of unknown origin before the onset of SE, (4) absence of psychobehavioral or memory alterations before the onset of SE, (5) absence of sustained orofacial–limb dyskinesias despite a profoundly decreased level of consciousness, and (6) symmetric brain magnetic resonance imaging (MRI) abnormalities.^26^, ^27^ Each feature represents 1 point; however, the first two clinical features are mandatory.^26^, ^27^

c‐NORSE score is a promising tool in low‐income countries, as it could help clinicians select treatments at the bedside, considering that the availability of AE testing is limited, and diagnosis is frequently delayed.^28^, ^29^ In this study, we aimed to describe a cohort of NORSE patients who were investigated for AE with TBA and CBA, and to evaluate the performance of the c‐NORSE score in a Brazilian series.

## 2 MATERIALS AND METHODS

We conducted a retrospective analysis of the Brazilian Autoimmune Encephalitis Network (BrAIN) database^28^ and selected patients tested for AE who presented SE or RSE from January 2017 to November 2023. Data on clinical presentation symptoms, paraclinical investigation, and treatment were compiled. This study was approved by the local ethics committee. Written informed consent was obtained from all legal representatives. We included both adults (≥18 years old) and pediatric patients (<18 years old) in this study.

All patients were screened for antineuronal abs using TBA for surface and intracellular abs. Positive samples were subsequently evaluated using commercial (Euroimmun) CBAs (anti‐N‐methyl‐D‐aspartate receptor [NMDAR], anti‐α‐amino‐3‐hydroxy‐5‐methyl‐4‐isoxazolepropionic acid receptor [AMPAR], anti‐γ‐aminobutyric acid type B receptor [GABA‐BR], anti‐leucine‐rich glioma‐inactivated 1 [LGI1], anti‐contactin‐associated protein‐like 2 [Caspr2], anti‐dipeptidyl‐peptidase‐like protein 6 [DPPX], and anti‐IgLON5), and immunoblot for anti‐glutamic acid decarboxylase 65 (GAD65) and high risk abs (former onconeural; Ravo PNS 14 Line Assay).^28^ Anti‐myelin oligodendrocyte glycoprotein (MOG), anti‐neurexin3alpha, anti‐metabotropic glutamate receptor 1 (mGluR1), anti‐mGluR5, and anti‐GABA‐AR abs were screened using in‐house live CBAs.

Additional information regarding NORSE criteria and the c‐NORSE score were obtained using a complementary electronic questionnaire. Information on prior epilepsy and other comorbidities, prodromal fever, presence of dyskinesia, memory or behavioral impairment prior to RSE, detailed electroencephalographic (EEG) and brain MRI results, genetic testing, and final diagnoses were compiled. All cases final diagnosis were reviewed by the authors.

Patients were classified as SE if they presented continuous seizures for ≥5 min or ≥2 seizures without complete recovery of consciousness in between.^30^ Only patients with prominent motor symptoms were included (convulsive SE). RSE was defined as SE refractory to parenteral therapy with ≥2 ASMs, including a benzodiazepine. According to a previously published consensus, NORSE was defined as RSE in a patient without previous epilepsy or other relevant neurological disorder.^1^ Continuous EEG monitoring was not considered a mandatory criterion, due to its low availability among Brazilian public hospitals.

Patients were classified as definite ab‐positive AE (definite AE) according to Graus and Cellucci criteria for adult and pediatric populations, respectively.^31^, ^32^ For probable seronegative AE (SN‐AE)^31^ we used the criteria of Graus: (1) compatible clinical syndrome (e.g., rapid progression of working memory deficits, altered mental status, or psychiatric symptoms); (2) absence of well‐characterized abs in serum and CSF; and (3) evidence of inflammatory changes in at least two of three tests: CSF analysis, brain MRI, and brain biopsy.

We classified as c‐NORSE patients with no identifiable etiology despite extensive workup, which included at least one brain MRI, EEG, CSF analysis with herpes polymerase chain reaction (PCR) test, and AE testing. We excluded the following: (1) patients not fulfilling the NORSE criteria, (2) nonconvulsive SE, and (3) those with history of relevant neurologic comorbidities (i.e., genetic conditions, stroke, or other structural brain lesions).

We calculated the c‐NORSE score, and patients were allocated into two groups: high score (HS), when score ≥ 5, or low score (LS), when score < 5. Clinical profile and abs status of both groups were compared using Mann–Whitney test, Fisher exact test, or chi‐squared test, when appropriate. We used a contingency table analysis for the sensitivity and specificity of the c‐NORSE score, and 95% confidence intervals (CIs) were estimated. R software (v.2023.12.1+402) was used for statistical analysis, and *p* < .05 was considered significant.

## 3 RESULTS

**FIGURE 1:**
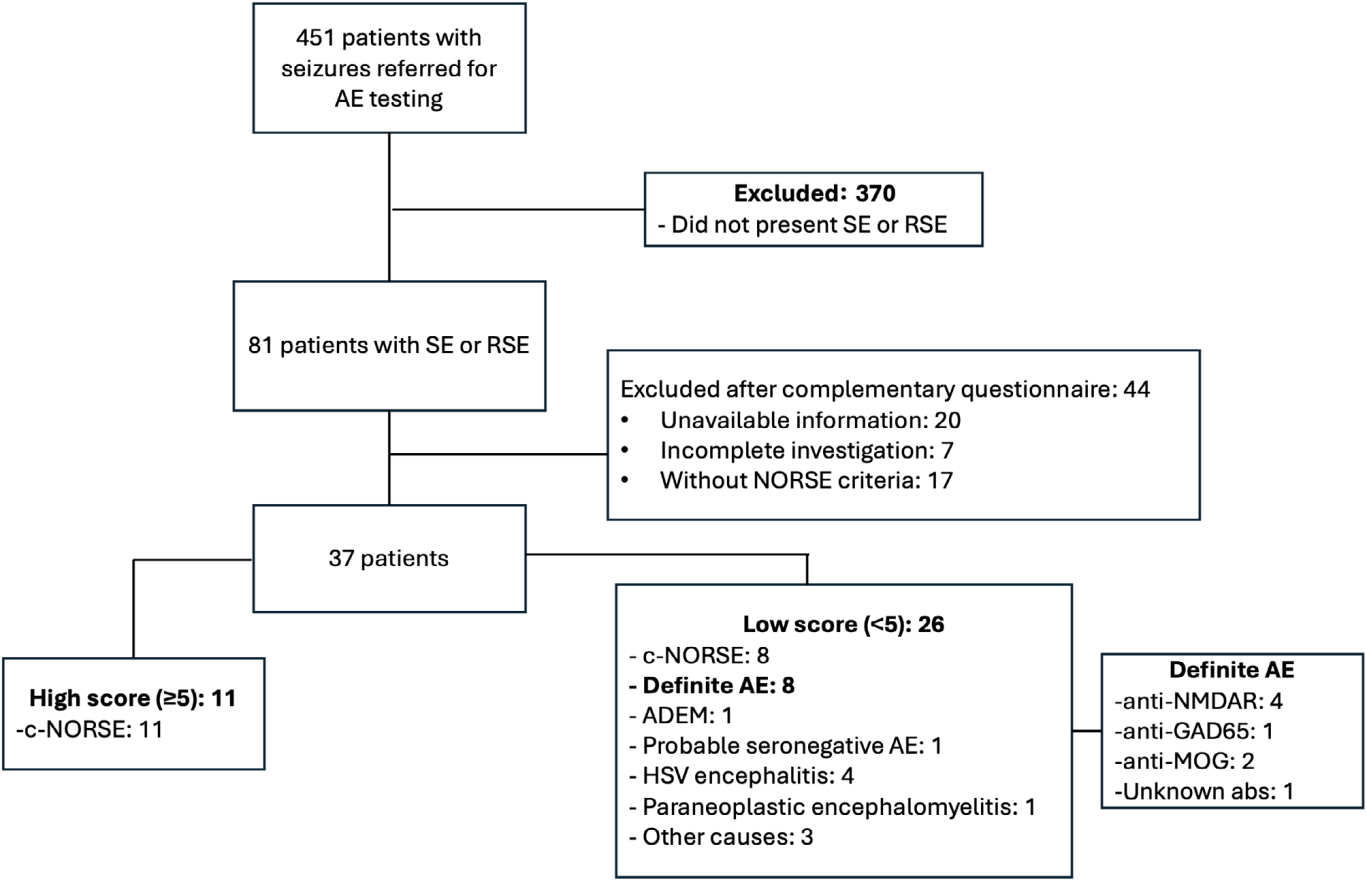
Flowchart of new onset refractory status epilepticus (NORSE) patient selection and cryptogenic NORSE (c‐NORSE) score calculation. Of 451 referred patients, 370 were excluded. Among 81 with status epilepticus (SE) and refractory SE (RSE), 40 were selected after further screening and divided into high and low c‐NORSE score groups. abs, antibodies; ADEM, acute disseminated encephalomyelitis; AE, autoimmune encephalitis; GAD65, glutamic acid decarboxylase 65; HSV, herpes simplex virus; MOG, myelin oligodendrocyte glycoprotein; NMDAR, N‐methyl‐D‐aspartate receptor.

We identified 451 patients with suspected seizures from the BrAIN databank; of those, 81 had SE or RSE. After complementary data acquisition for NORSE criteria, 37 patients were selected. Forty‐four patients were excluded due to loss of follow‐up, incomplete investigation, or lack of NORSE criteria. The study flowchart is summarized in Figure 1.

### 3.1 Demographic and clinical characteristics and complementary investigation of NORSE patients

Overall, NORSE patients were young, and pediatric patients represented 62% (23/37) of the cohort. Nearly half of the patients (49%) were female. Table 1 summarizes clinical and complementary investigation data. Eight patients (22%) had AE, and 19 (51%) were classified as c‐NORSE. Approximately 84% of the patients received immunotherapies such as methylprednisolone, IVIG, or plasmapheresis. The frequency of each ASM is described in Table S1 (Supplementary File).

**TABLE 1:** Demographic and clinical characteristics and complementary investigation of new onset refractory SE patients (*N* = 37).

Variable	Value
**Clinical and demographic variables**	
Median age, years (IQR)	15 (5–23)
Female, *n* (%)	18 (49%)
Fever, *n* (%)	23 (62%)
Cognitive/behavioral symptoms, *n* (%)	26 (70%)
Dyskinesia, *n* (%)	14 (38%)
Median days of SE duration (range)	9 (1–120)
Neoplasia, *n* (%)	1 (3%)
**Complementary investigation**	
Median CSF WBC count, cells/mL (IQR)	5 (1–13)
OCB present, *n* (%)	2 (14%)
Abnormal MRI, *n* (%)	22 (59%)
Genetic testing, *n* (%)	4 (11%)
Patients evaluated with cEEG, *n* (%)	10 (26%)
Definite AE, *n* (%)	8 (22%)
**Treatment** *n* (%)	31 (84%)
IV methylprednisolone *n* (%)	30 (81%)
IVIG *n* (%)	20 (54%)
Plasmapheresis *n* (%)	5 (13%)
Rituximab *n* (%)	3 (8%)
Cyclophosphamide *n* (%)	4 (11%)
Tocilizumab *n* (%)	1 (3%)

Abbreviations: AE, autoimmune encephalitis; cEEG, continuous electroencephalography; CSF, cerebrospinal fluid; IQR, interquartile range; IV, intravenous; IVIG, IV immunoglobulin; MRI, magnetic resonance imaging; OCB, oligoclonal band; SE, status epilepticus; WBC, white blood cell.

Among pediatric patients, we found that two patients had anti‐MOG and two had anti‐NMDAR abs, resulting in a seropositivity rate of four of 23 (17%). c‐NORSE represented 16 of 23 (69.5%) pediatric patients. Among adults, the seropositivity rate was four of 14 (29%; two anti‐NMDAR, one anti‐GAD65, and one unknown ab), and c‐NORSE represented 21% (3/14). Pediatric patients more frequently presented fever of unknown origin preceding RSE (FIRES; pediatric 78% vs. adults 36%, *p* = .01) and c‐NORSE (pediatric 69% vs. adults 21%, *p* = .006; Table 2).

**TABLE 2:** Comparison of pediatric and adult NORSE patients from the Brazilian cohort.

Variable	Pediatric, *n* = 23	Adults, *n* = 14	*p*
Median age, years (IQR)	7 (3–12)	24 (21–42)	
Female, *n* (%)	10 (43%)	8 (57%)	.42
Prodromal fever, *n* (%)	18 (78%)	5 (36%)	.01*
Memory/behavioral symptoms, *n* (%)	16 (70%)	10 (71%)	1
Orofacial–limb dyskinesia, *n* (%)	11 (48%)	3 (21%)	.10
Abnormal MRI, *n* (%)	15 (65%)	7 (50%)	.36
Bilateral and symmetric MRI findings, *n* (%)	10 (67%)	4 (57%)	1
Median CSF cells, *n* (IQR)	4 (1–8)	9 (2–22)	.10
Definite AE, *n* (%)	4 (17%)	4 (29%)^*^	.44
High c‐NORSE score, *n* (%)	9 (39%)	2 (14%)	.15
c‐NORSE, *n* (%)	16 (69%)	3 (21%)	.006*

Abbreviations: AE, autoimmune encephalitis; c‐NORSE, cryptogenic NORSE; CSF, cerebrospinal fluid; IQR, interquartile range; MRI, magnetic resonance imaging; NORSE, new onset refractory status epilepticus.

^*^ *p* < 0.05

Among the patients with anti‐MOG abs, Patient 1 was a 3‐year‐old male who developed ataxia, aphasia, reduced level of consciousness, and RSE. Brain MRI revealed multiple T2/fluid‐attenuated inversion recovery (FLAIR) hyperintensities in cerebral cortex and white matter, and anti‐MOG was positive in serum (titer 1:160) and negative in CSF. Patient 2, also a 3‐year‐old male, developed ataxia and RSE, with MRI showing cortical T2/FLAIR hyperintensities in cerebellum and left temporal and frontal cortex. Testing for anti‐MOG abs showed a titer of 1:40 (below high‐titer cutoff of 1:160); however, testing was performed after administration of immunotherapy that might have reduced the MOG‐IgG. Both patients had a clinical phenotype of acute disseminated encephalomyelitis (ADEM) and fulfilled MOGAD (MOG ab‐associated disease) criteria, according to a recent international panel.^33^ The patient with anti‐GAD65 abs was an 18‐year‐old male who presented with generalized seizures, neuropsychiatric symptoms, and RSE, which improved after IVIG treatment. Although serum titers were unavailable (retrospective chart review), anti‐GAD65 was confirmed in CSF using TBA and immunoblot, supporting the diagnosis of anti‐GAD65 encephalitis.

The one patient harboring an unknown ab presented a TBA with a strong and specific synaptic staining pattern in both serum and CSF. Additionally, staining of live hippocampal neurons was strongly positive. These findings suggest the presence of antineuronal surface abs. Subsequent commercial CBAs (Euroimmun) and in‐house GABA‐AR, AMPAR, and mGluR5 were negative, and further efforts to identify the target antigen using immunoprecipitation were unsuccessful. One adult patient fulfilled the SN‐AE criteria, presenting with subacute behavioral symptoms, RSE, pleocytosis, and asymmetrical cortical lesions on brain MRI.

### 3.2 Application of c‐NORSE score, sensitivity, and specificity

Eleven (30%) patients were assigned to the HS group and 26 (70%) to the LS group. HS patients had more frequent bilateral and symmetric MRI findings (HS 100% vs. LS 43%, *p* = .018) and less frequent memory or behavioral symptoms (HS 27% vs. LS 89%, *p* = .001). There was no difference between groups regarding gender, prodromic fever, and orofacial–limb dyskinesia (Table 3).

**TABLE 3:** Comparison of clinical and cryptogenic new onset refractory status epilepticus score variables between LS and HS groups.

Variable	LS group [score < 5], *n* = 26	HS group [score ≥ 5], *n* = 11	*p*
Median age, years (IQR)	16 (6–24)	10 (5–16)	.26
Female, *n* (%)	13 (50%)	5 (45%)	.80
Prodromal fever, *n* (%)	14 (54%)	9 (82%)	.15
Memory or behavioral symptoms, *n* (%)	23 (89%)	3 (27%)	.001*
Orofacial–limb dyskinesia, *n* (%)	12 (46%)	2 (18%)	.15
Bilateral and symmetric MRI findings, *n* (%)	6 (43%)	8 (100%)	.018*
Median CSF cells, *n* (IQR)	6 (1–14)*	4 (2–6)	.36
Definite AE, *n* (%)	8 (31%)	0	.07

Abbreviations: AE, autoimmune encephalitis; CSF, cerebrospinal fluid; HS, high score; IQR, interquartile range; LS, low score; MRI, magnetic resonance imaging.

^*^ p < 0.05

Patients in the LS group had the following final diagnoses: c‐NORSE (*n* = 8), definite AE (*n* = 8), SN‐AE (*n* = 1), herpes simplex virus (HSV) encephalitis (*n* = 4), ADEM (*n* = 1), paraneoplastic encephalomyelitis associated with lymphoma (*n* = 1), immunodeficiency with opportunistic infection (*n* = 1), neuropsychiatric systemic lupus erythematosus (SLE; *n* = 1), and immediate postoperative period in a patient with Parkinson disease (*n* = 1; Figure 1).

All patients from the HS group remained as c‐NORSE, despite extensive investigation. The sensitivity of c‐NORSE score ≥ 5 was 57.9% (95% CI = 36%–80%), and the specificity was 100% (95% CI = 84%–100%). Table S2 presents the contingency table analysis for sensitivity and specificity.

## 4 DISCUSSION

We found that the c‐NORSE score in a Brazilian cohort had a sensitivity of 57.9% (95% CI = 36%–80%), contrasting with the original report of 93.9%.^27^ This finding indicates that sensitivity may vary depending on the categorization of the final diagnosis of each patient. For instance, the pivotal study from Yanagida et al. classified the etiology of NORSE from 11 patients fulfilling the possible AE criteria with negative abs as miscellaneous etiology, indicating that they had a possible cause for RSE. However, recent data showed that some patients fulfilling the possible AE criteria with negative abs actually had primary psychiatric diseases, neurodegenerative diseases, Creutzfeldt–Jakob disease, and brain neoplasms.^16^, ^28^, ^34^, ^35^, ^36^ Therefore, possible AE is a term that should not be considered as a cause of NORSE. When applying this conceptual framework, one might assume that among those 11 patients with possible AE some were c‐NORSE (there was no cause for the RSE), which would lead to a lower sensitivity of the c‐NORSE score. Our study classified patients with possible AE with negative abs as c‐NORSE, when SN‐AE was excluded, and the final diagnosis was unknown after complementary investigation and retrospective chart review.^37^

In this study, definite AE was the most common identified cause of NORSE, with 22% of the patients harboring antineuronal/antiglial abs. Our data are relevant as (1) we tested the cohort with two complementary techniques (TBA and CBA) in paired serum/CSF for antineuronal abs, (2) we only defined as definite AE those patients harboring abs with established pathogenicity, and (3) we tested all pediatric cases for anti‐MOG abs. By following current testing recommendations, our AE rate among pediatric NORSE (17%) was considerably higher than the 3%–7% reported in other cohorts,^3^, ^6^, ^11^ and we detected anti‐NMDAR and anti‐MOG abs, which are the most common in pediatric AE.^32^, ^38^, ^39^ In pediatric patients with suspected AE, anti‐MOG should be screened,^38^, ^40^ and although anti‐MOG abs have already been associated with SE,^33^ and even with NORSE,^41^ we believe our findings support the inclusion of anti‐MOG abs in routine diagnostic testing of pediatric NORSE patients.

Despite the smaller sample size of our study, we have observed some misclassification and misinterpretation of ab results in prior studies; patients with neuropsychiatric SLE, with anti‐voltage‐gated potassium channel complex, with antistriational abs (with unclear clinical significance),^34^, ^42^ and with SREAT (steroid‐responsive encephalopathy associated with autoimmune thyroiditis), a diagnosis that requires systematically ruling out of AE,^43^ were all classified as AE. Our results confirm that the most identified cause of NORSE is AE and that formal published criteria should be observed to prevent misdiagnosis.

In our study, the overall rate of c‐NORSE was 51%, which was higher among children (pediatric 69% vs. adults 21%, *p* = .006), as previously described in the literature. Additionally, most pediatric NORSE cases were FIRES (pediatric 78% vs. adults 36%, *p* = .01), favoring that variable mechanisms may lead to RSE in children.^3^, ^4^ However, the frequency of c‐NORSE in the adult cohort (21%) was notably lower than reported in previous studies (>50%).^2^, ^7^ This discrepancy is likely due to selection bias, as our cohort was derived from an AE database, which may underrepresent c‐NORSE cases.

One critical finding of our study was the specificity of 100% (95% CI = 84%–100%) of the c‐NORSE score, which aligns with the pivotal validation study^27^ and suggests that it is a valuable tool at the bedside for c‐NORSE identification. Our results indicate that when the score is ≥5, no apparent cause for NORSE is identified. This aligns with two previous studies involving patients with c‐NORSE and negative antineuronal abs, suggesting that the pathophysiology is not ab‐mediated.^26^, ^44^ Although we have a small sample size of patients, our findings favor that the score may help identify those who would benefit the most from AE investigation (i.e., those with low score), which is essential for low‐income countries with limited access to AE testing. Nevertheless, future studies are needed to confirm this finding.

Another interesting finding of this study was the final etiologies of NORSE among patients with c‐NORSE score < 5. In this specific group, the final diagnosis was AE, infectious encephalitis, and autoimmune diseases. Such findings highlight the need for extensive complementary investigation in NORSE patients, particularly in this group. An international consensus recommends that beyond AE testing, NORSE patients should undergo comprehensive rheumatologic and infectious evaluation, and screening for inborn errors of metabolism in young children.^24^ In selected cases, blood and CSF samples should be stored for further cytokine and genetic analysis.^24^

This study has some limitations. Its retrospective design and lack of follow‐up data introduced heterogeneity into the complementary investigations, despite mandatory inclusion criteria such as brain MRI, CSF analysis, and herpes PCR testing. As a multicenter study conducted in a resource‐limited setting, testing for additional viral agents beyond HSV and toxicological evaluations was unavailable. Our databank was designed for patients suspected of having AE, which may have led to selection bias and slight epidemiological discrepancies compared to other NORSE series. For example, adult NORSE patients in our cohort were younger than those reported by Gaspard et al.,^2^ whose study showed a bimodal age distribution peaking at 28.5 and 65.5 years. Pediatric patients in our study were older than in prior reports (median age = 7 years). Genetic testing was conducted in only 10% of cases, primarily due to limited access, potentially affecting the reported c‐NORSE rate. This is significant, as previous studies found that nearly 25% of pediatric NORSE patients had genetic or metabolic disorders.^11^ Furthermore, continuous EEG monitoring was unavailable for several patients, which may have influenced ASM titration and the estimation of NORSE duration. Despite these challenges, we believe our data accurately reflect the realities of clinical practice in resource‐limited settings.

In summary, after performing TBA and CBA for antineuronal and antiglial ab detection, we found that 22% of NORSE patients had AE. A c‐NORSE score ≥ 5 had a specificity of 100% (95% CI = 84%–100%) for identifying cryptogenic NORSE cases, whereas a score < 5 indicated that additional investigation should be ordered. c‐NORSE score may help clinicians to select treatments, and it is a useful tool for bedside NORSE evaluation in low‐income countries, where access to abs testing is limited. Applying AE diagnostic criteria and complementary investigation may influence the score sensitivity, especially in resource‐limited settings. These findings must be further confirmed in other populations.

## AUTHOR CONTRIBUTIONS

*Substantial contributions to the design or development of the study:* João Henrique Fregadolli Ferreira, Anna Maria Gomes, Ana Carolina Zetehaku, Taissa Ferrari Marinho, Fabio Fieni Toso, Caio César Diniz Disserol, Mariana Braatz Krueger, Mara Lúcia Schmitz Ferreira Santos, Aline Freire Borges Juliano, Tércio Luz Barbosa, Letícia Januzi de Almeida Rocha, Fernando Tenório Gameleira, Renata Barbosa Paolilo, Vanessa Daccach, João Pedro Izidoro Gomes, Katia Lin, Adaucto Wanderley da Nóbrega Jr., Emily Lima Marmentini, Eduardo Ferracioli Fusão, Anderson de Cuffa, Georgia Lelis Aranha Tavares, Ronaldo Maciel Dias, Sabrina Stephanie Lana Diniz, Fábio Siquineli, Pedro Braga‐Neto, Paulo Ribeiro Nobrega, Lorena Pitombeira Sanders, Fernanda Martins Maia Carvalho, Renata Brasileiro Reis Pereira, Eduardo Sousa de Melo, Adélia Maria de Miranda Henriques‐Souza, Clecio de Oliveira Godeiro Jr., Omar Gurrola Arambula, Filipe Nolasco de Souza e Silva, Karla Oliveira Couto, Dayane Danieli, Katia Werneck Seitz, Alessandra Dellavance, Luis Eduardo Coelho Andrade, Luis Otavio Caboclo, and Livia Almeida Dutra. *Substantial contributions to the collection, analysis, and interpretation of data:* João Henrique Fregadolli Ferreira, Anna Maria Gomes, Ana Carolina Zetehaku, Taissa Ferrari Marinho, Fabio Fieni Toso, Caio César Diniz Disserol, Mariana Braatz Krueger, Mara Lúcia Schmitz Ferreira Santos, Aline Freire Borges Juliano, Tércio Luz Barbosa, Letícia Januzi de Almeida Rocha, Fernando Tenório Gameleira, Renata Barbosa Paolilo, Vanessa Daccach, João Pedro Izidoro Gomes, Katia Lin, Adaucto Wanderley da Nóbrega Jr., Emily Lima Marmentini, Eduardo Ferracioli Fusão, Anderson de Cuffa, Georgia Lelis Aranha Tavares, Ronaldo Maciel Dias, Sabrina Stephanie Lana Diniz, Fábio Siquineli, Pedro Braga‐Neto, Paulo Ribeiro Nobrega, Lorena Pitombeira Sanders, Fernanda Martins Maia Carvalho, Renata Brasileiro Reis Pereira, Eduardo Sousa de Melo, Adélia Maria de Miranda Henriques‐Souza, Clecio de Oliveira Godeiro Jr., Omar Gurrola Arambula, Filipe Nolasco de Souza e Silva, Karla Oliveira Couto, Dayane Danieli, Katia Werneck Seitz, Alessandra Dellavance, Luis Eduardo Coelho Andrade, Luis Otavio Caboclo, and Livia Almeida Dutra. *Substantial contributions to the writing of the article or to its critical revision:* João Henrique Fregadolli Ferreira, Anna Maria Gomes, Caio César Diniz Disserol, Katia Lin, Adaucto Wanderley da Nóbrega Jr., Paulo Ribeiro Nobrega, Fernanda Martins Maia Carvalho, Luis Eduardo Coelho Andrade, Luis Otavio Caboclo, and Livia Almeida Dutra. *Substantial contributions to the approval of the final version:* João Henrique Fregadolli Ferreira, Anna Maria Gomes, Caio César Diniz Disserol, Katia Lin, Adaucto Wanderley da Nóbrega Jr., Paulo Ribeiro Nobrega, Fernanda Martins Maia Carvalho, Luis Eduardo Coelho Andrade, Luis Otavio Caboclo, and Livia Almeida Dutra.

## CONFLICT OF INTEREST STATEMENT

L.A.D. has received two grants from the Fleury laboratory for the Brain Project and Brain Registry project. The remaining authors have no conflict of interest.

## ETHICS STATEMENT

All patients provided consent according to the local ethic committee. We confirm that we have read the Journal's position on issues involved in ethical publication and affirm that this report is consistent with those guidelines.

## Supporting information


Table S1.

## Data Availability

The data that support the findings of this study are available on request from the corresponding author. The data are not publicly available due to privacy or ethical restrictions.
