# Supplementary material for: Performance of cryptogenic new onset refractory status epilepticus score in a Brazilian cohort after testing for antineuronal antibodies with tissue‐based and cell‐based assays
Source: Epilepsia. 2025 May 2;66(7):2285–94. doi: 10.1111/epi.18374 (PMC12291003; doi:10.1111/epi.18374)
Supplement: Supplementary file 1 — Table S1. [file EPI-66-2285-s001.docx]

| Table S1 – Anti-seizure medications in Brazilian NORSE patients | |  |
| --- | --- | --- |
| **ASM** | ***n* = 32** |  |
| Midazolam *n* (%) | 29 (88%) |  |
| Clobazam *n* (%) | 13 (39%) | |
| Diazepam *n* (%) | 14 (42%) | |
| Phenytoin *n* (%) | 29 (88%) | |
| Phenobarbital *n* (%) | 26 (79%) |  |
| Oxcarbazepine *n* (%) | 1 (3%) | |
| Valproic acid *n* (%) | 19 (57%) | |
| Topiramate *n* (%) | 13 (39%) | |
| Levetiracetam *n* (%) | 17 (51%) | |
| Propofol *n* (%) | 10 (30%) | |
| Lacosamide *n* (%) | 4 (12%) | |
| Lamotrigine *n* (%) | 3 (9%) | |
| Thiopental *n* (%) | 14 (42%) | |
| Ketamine *n* (%) | 11 (33%) | |
| Cannabidiol *n* (%) | 3 (9%) | |

Legend: ASM: anti-seizure medication

Table S2 – Contingency table of the c-NORSE score in Brazilian NORSE patients

| **c-NORSE** | | **NORSE with identifiable etiology** | | |  |
| --- | --- | --- | --- | --- | --- |
| High score | 11 | | 0 |  | |
| Low score | 8 | | 18 |  | |
| Legend: c-NORSE: cryptogenic new-onset refractory status epilepticus | | | | | |
